# Abrikossoff Tumor Clinically Mimicking Carcinoma in Accessory Axillary Breast Tissue

**DOI:** 10.7759/cureus.21733

**Published:** 2022-01-30

**Authors:** Lyronne Olivier, Vijay Naraynsingh, Dale Hassranah, Christopher Cassim

**Affiliations:** 1 General Surgery, Sangre Grande Regional Hospital, Sangre Grande, TTO; 2 General Surgery, Medical Associates Hospital, St. Joseph, TTO; 3 Interventional Radiology, Sangre Grande Regional Hospital, Sangre Grande, TTO

**Keywords:** accessory breast tissue, myoblastoma, granular cell tumor, general surgery, breast imaging, breast surgery, benign breast condition

## Abstract

Abrikossoff tumors are rare benign soft-tissue lesions also known as granular cell tumors (GCT). The histogenesis of these tumors was initially considered to be myogenic but recent studies have revealed a neuroectodermal origin. GCTs of the breast may mimic breast carcinoma based on the triad of radiological, clinical, and pathological features. This hallmark trait lends to the misdiagnosis of these tumors and their subsequent inappropriate management. We report a rare case of a 28-year-old female patient with an accessory axillary breast GCT. The diagnosis, histogenesis, and management of Abrikossoff tumors of the breast are discussed.

## Introduction

Weber and Virchow first mentioned granular cell tumors (GCTs) of the tongue in 1854 [1]. Abrikossoff fully described GCTs as "myoblastoma" of the tongue in 1926 [2], and then specifically breast GCT (B-GCT) in 1931 [3]. GCTs may arise from the subdermal, intradermal, or submucosal layers of the affected tissue. The initial histogenesis of these tumors was considered to be myogenic in nature; however, recent immunohistochemistry (IHC) and ultrastructural analysis bring another perspective to this theory.

Abrikossoff tumors of the breast are rare benign GCTs. These lesions originate from the interlobular stroma of the breast [4-6]. They may present with the characteristic clinical, radiological, and pathological features similar to that of breast carcinoma. The classic clinical features of invasive breast cancer, such as a hard mass with ill-defined irregular borders, poor mobility with skin tethering, and dimpling, are vividly apparent in these tumors. Thus, misdiagnosis of these tumors as cancer may lead to the inappropriate aggressive management of the patients with significant physical and psychological adverse outcomes. Therefore, the tenants of triple assessment, a thorough medical history and physical examination, and radiological workup followed by a breast biopsy, remain an integral clinical blueprint.

B-GCTs account for 5-15% of GCTs [6]. However, most of these tumors are benign, and less than 1% of GCTs are found to have the histological and clinical features of malignancy [4]. B-GCTs have a high prevalence in the upper inner quadrant of the breast. This affiliation is thought to be associated with its origin from the cutaneous branches of the supraclavicular nerves, further clinically implicating the histogenesis theory of neuroectodermal origin [6].

Other sites in the breast have also been described in the literature. However, the accessory breast tissue is considered to be a relatively rare site for B-GCTs. A literature search revealed only one case of accessory breast tissue GCT, further exemplifying the rarity of our case [7].

## Case presentation

A 28-year-old female patient with African ancestry presented to the surgical outpatient clinic with a history of an axillary mass for over 10 years; this slowly increased in size during this time. The triple assessment was implemented in this patient's initial evaluation.

**Figure 1:**
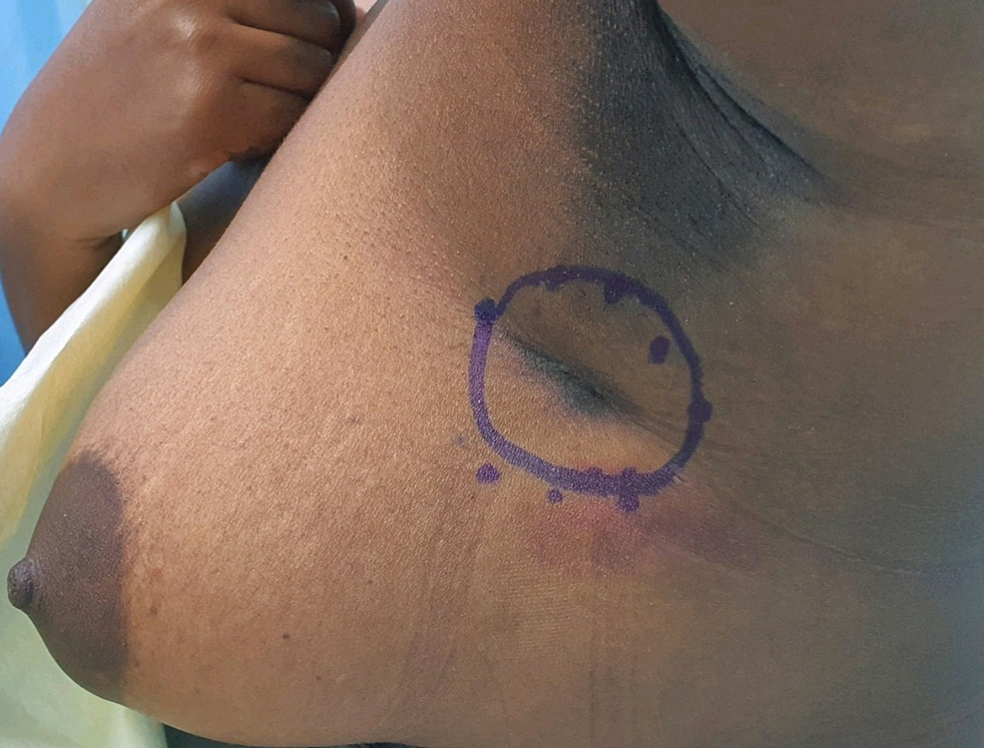
The clinical appearance of axillary Abrikossoff tumor with associated pseudoepitheliomatous hyperplasia of the skin.

On examination, this mass was fixed to the underlying muscle and tethered to the skin with associated hyperpigmentation. The lesion was 3 x 3 cm in size with ill-defined irregular borders (Figure 1).

**Figure 2:**
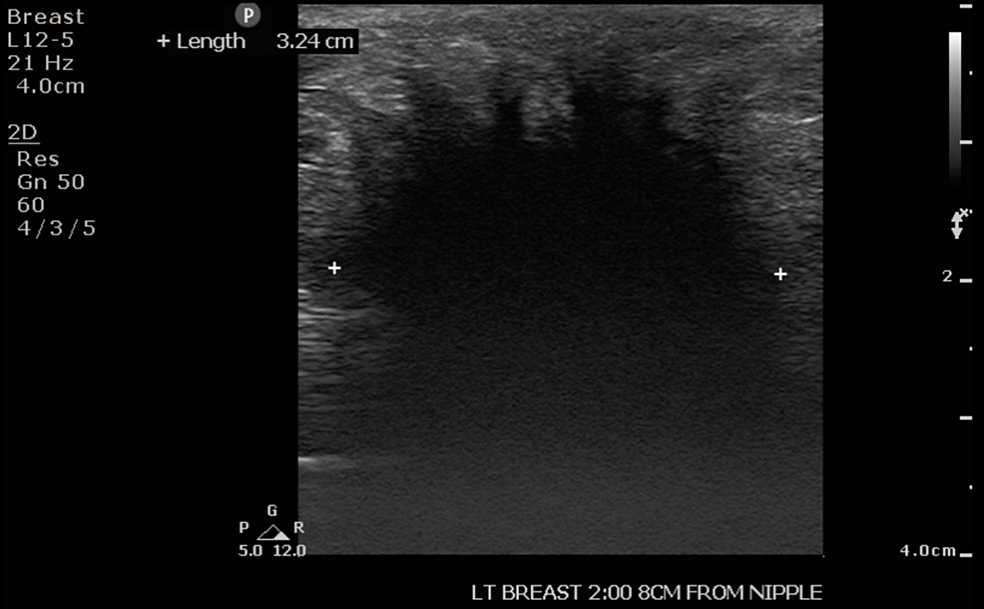
Ultrasound image of the left axillary accessory breast granular cell tumor.

**Figure 3:**
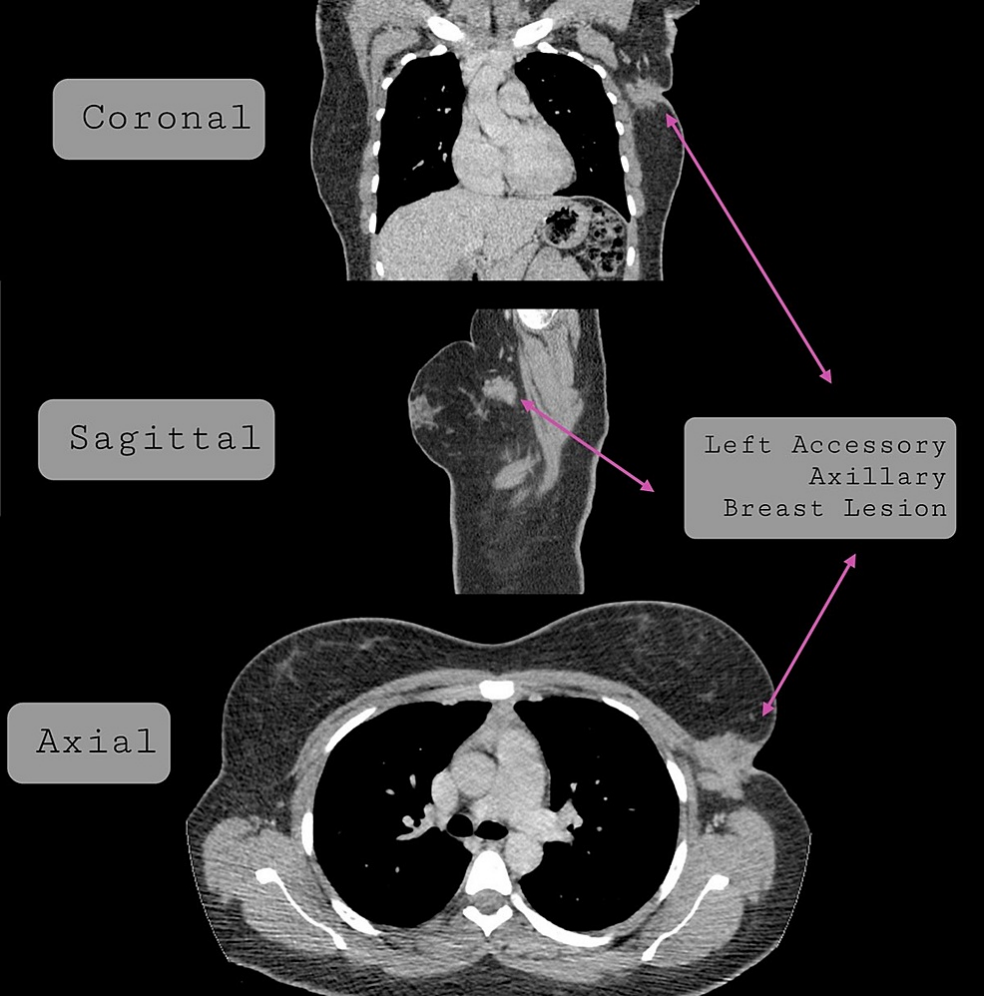
Computed tomography images of accessory axillary breast lesion.

Ultrasound of the axilla revealed a Breast Imaging-Reporting and Data System (BI-RADS) 5, ill-defined, hypoechoic mass measuring 3.5 cm in size (Figure 2). Computed tomography (CT) scan of the chest with contrast demonstrated a 4.0 x 3.6 cm, irregular, ill-defined mass with spiculated borders associated with skin retraction and involvement of the lateral border of left pectoralis major muscle (Figure 3). Morphologically normal lymph nodes were observed in both ultrasound and CT scans. Each modality of imaging documented a highly suspicious malignant lesion and an ultrasound-guided biopsy was subsequently performed.

**Figure 4:**
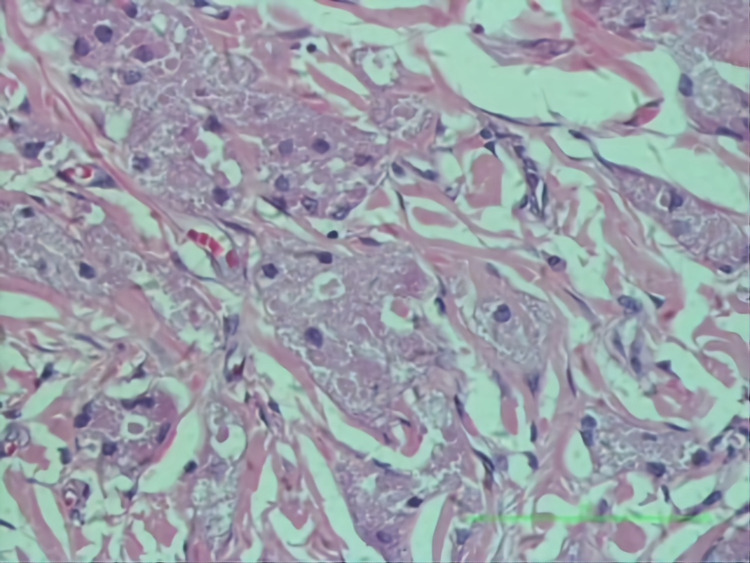
Granular cell tumor composed of ribbons and syncytial nests of polygonal cells possessing abundant, eosinophilic, granular cytoplasm and small dense nuclei, separated by thin collagenous bands.

**Figure 5:**
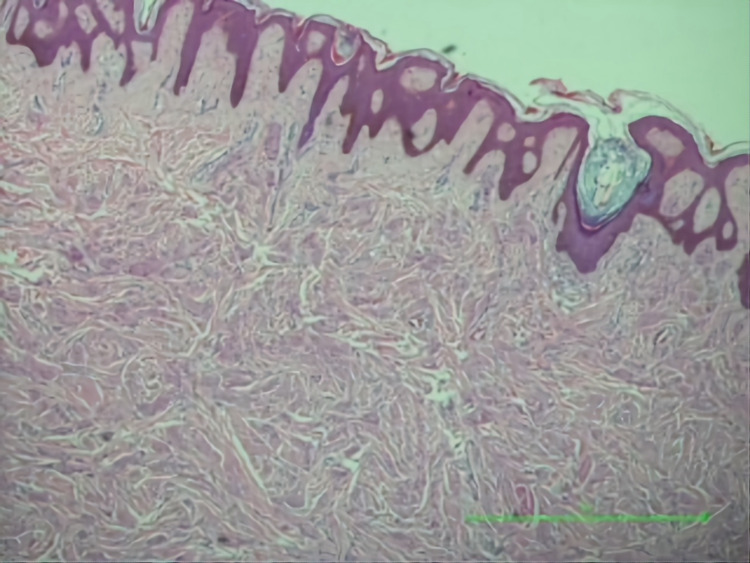
Granular cells infiltrating the upper dermis associated with focal pseudoepitheliomatous hyperplasia of the overlying epidermis.

The pathological report for the needle core biopsy was inconclusive but suggested sclerotic breast parenchymal tissue with foamy inflammatory changes. Wide local excision was performed under general anesthesia. The axillary lesion was found to be separate from the breast parenchyma and in close proximity to the pectoralis and the underlying serratus anterior fascia. The long thoracic nerve of Bell was identified and preserved. Final excisional histology revealed a benign B-GCT (Figure 4) with margins free of tumor and associated with pseudoepitheliomatous hyperplasia (PEH) of the overlying epidermis (Figure 5).

## Discussion

Abrikossoff tumors of the breast are rare benign lesions that can mimic breast carcinoma. The characteristics of GCTs create clinical perplexity by their inert cirrhosis nature. GCT's ability to locally invade the superficial tissue creates the infiltrative appearance of this lesion. Therefore, it is of paramount importance for the preoperative histopathological assessment of these breast tumors prior to overzealous surgical management, which could result in unwarranted mastectomy and axillary surgical evaluation.

B-GCTs usually present as a solitary mass. However, 10% of cases have multiple GCTs and are associated with the autosomal dominant Noonan syndrome [8]. This syndrome is associated with short stature, heart defects, unusual facial features, bleeding disorder, leukemia, skeletal malformation, and the predilection of multiple GCTs. B-GCTs have been found to affect predominantly perimenopausal women of African ancestry [9]. The first account of B-GCTs in Trinidad and Tobago was described in a series of five patients by Naraynsingh et al. in 1985 [10]. Middle-aged perimenopausal African women were the predominant epidemiological finding noted in this study [10].

Radiologically, B-GCTs have similar features to invasive carcinoma [4]. Mammographically, spiculation, irregular borders, isodensity, and stellation may be observed. On ultrasound, malignant features of the heterochronic spiculated lesion, shadowing, and irregular borders without or with peripheral vascularity may be seen [4]. MRI scan also lacks specificity in distinguishing GCTs from invasive breast lesions [4]. Therefore, preoperative imaging has significant diagnostic shortcomings of these lesions. The final appraisal should be guided by pathological assessment.

Radiological imaging may reveal the involvement of the skin. Clinically, this may be identified by the presence of PEH of the epidermis [11]. This presentation represents a reactive change to the underlying tumor and not an actual direct tumor extension. Therefore, this may be a clinical marker for the presence of the underlying B-GCTs and not considered an up-staging feature as observed in breast carcinoma staging.

Abrikossoff "myoblastoma" was initially thought to be myogenic in its origin [2]. However, the identification of certain proteins via IHC depicts an alternate neuroectodermal differentiation. This is likely arising from Schwann cells of the peripheral nervous system [8]. Tissue samples positive for S-100, CD68 (KP-1), and neuron-specific enolase (NSE), as well as cytokeratin, estrogen, and progesterone receptor negativity, are considered confirmatory for the diagnosis of GCTs [9].

The S-100 protein is abundantly produced by the Schwann cell during times of distress such as the presence of cancer cells or injury [7]. These proteins result in the chemotaxis of Schwann cells to the cytoplasm and cumulative increase release of S-100. This IHC marker is sensitive for GCTs but it is not specific. A total of 10% of breast malignancy also demonstrates S-100 positivity as well as melanocytes and fat tissue cells [12]. Breast malignancy can be differentiated by cytokeratin positivity [4].

CD68 protein stains positively in 90% of GCT, demonstrating the presence of marked lysosomal activity [13]. The pathognomic granular appearance of GCTs originates from a similar mechanism as external folding of myelin sheath formation around the nerves. Internal membrane foldings in B-GCTs undergo phagocytosis, depicting the increased and marked lysosomal activity [14]. This resultant granular appearance of the cytoplasm stains periodic acid Schiff (PAS) was positive because of the intracytoplasmic lysosomes abundance.

The majority of B-GCTs are benign but less than 1% may demonstrate histological and clinical features of malignancy [4]. Fanburg-Smith et al. described the histological criteria for the diagnosis of malignant GCTs as necrosis, vesicular nuclei with large nucleoli, high nuclear to cytoplasmic ratio, pleomorphism, tumor cells spindling, and increased mitotic rate greater than 2 mitoses per 10 high power field [15]. If a patient has one or two features, the pathological diagnosis of the atypical lesion is given.

However, based on this classification, the presence of three or more criteria labels this specimen malignant. Conversely, GCTs are considered benign in the absence of all features. Contrary to this histological definition of malignancy, other experts may consider lesions malignant without the aforementioned criteria. The clinical presentation of malignant diseases such as metastatic spread or regional axillary lymph nodal involvement may confirm a malignant diagnosis in atypical patients.

The surgical management of benign B-GCTs is wide local excision with free margins. Benign GCTs do not metastasize to the regional nodes; hence, axillary staging is not required. Patients require post-surgical surveillance for approximately 10 years since the risk of local recurrence is approximately 2-8% with negative surgical margins and more than 20% with positive surgical margins [12]. Therefore, assessment and reporting of surgical margins are crucial to lessen recurrences [16]. These tumors generally have a very good prognosis, and adjuvant therapy in the form of chemotherapy or radiation has shown no survival benefit [12].

## Conclusions

Abrikossoff breast tumors are rare benign GCTs with suspicious characteristic features of invasive breast cancer observed clinically and radiologically. However, the tenets of the "triple assessment" hold fast in the management of all breast and axillary lesions despite the overwhelming pathological characteristics. This case report highlights the importance of such presentation because the inappropriate clinical diagnosis may lead to unwarranted mastectomy and axillary surgery for a benign lesion.
